# Circadian rhythm disruption-mediated downregulation of Bmal1 exacerbates DSS-induced colitis by impairing intestinal barrier

**DOI:** 10.3389/fimmu.2024.1402395

**Published:** 2024-06-04

**Authors:** Zhongchao Zhang, Wanneng Li, Xu Han, Dean Tian, Wei Yan, Mei Liu, Li Cao

**Affiliations:** Department of Gastroenterology and Hepatology, Tongji Hospital, Tongji Medical College, Huazhong University of Science and Technology, Wuhan, China

**Keywords:** ulcerative colitis, Bmal1, apoptosis, intestinal barrier, autophagy

## Abstract

**Background:**

Circadian rhythm disruption (CRD) is thought to increase the risk of inflammatory bowel disease. The deletion of Bmal1, a core transcription factor, leads to a complete loss of the circadian rhythm and exacerbates the severity of dextran sodium sulfate (DSS)-induced colitis in mice. However, the underlying mechanisms by which CRD and Bmal1 mediate IBD are still unclear.

**Methods:**

We used a CRD mouse model, a mouse colitis model, and an *in vitro* model of colonic epithelial cell monolayers. We also knocked down and overexpressed Bmal1 in Caco-2 cells by transfecting lentivirus *in vitro*. The collected colon tissue and treated cells were assessed and analyzed using immunohistochemistry, immunofluorescence staining, quantitative reverse transcription-polymerase chain reaction, western blot, flow cytometry, transmission electron microscopy, and terminal deoxynucleotidyl transferase-mediated dUTP nick-end labelling staining.

**Results:**

We found that CRD mice with downregulated Bmal1 expression were more sensitive to DSS-induced colitis and had more severely impaired intestinal barrier function than wild-type mice. Bmal1-/- mice exhibited more severe colitis, accompanied by decreased tight junction protein levels and increased apoptosis of intestinal epithelial cells compared with wild-type mice, which were alleviated by using the autophagy agonist rapamycin. Bmal1 overexpression attenuated Lipopolysaccharide-induced apoptosis of intestinal epithelial cells and impaired intestinal epithelial cells barrier function *in vitro*, while inhibition of autophagy reversed this protective effect.

**Conclusion:**

This study suggests that CRD leads to the downregulation of Bmal1 expression in the colon, which may exacerbate DSS-induced colitis in mice, and that Bmal1 may serve as a novel target for treating inflammatory bowel disease.

## 1 Introduction

Inflammatory bowel disease (IBD), which includes Crohn’s disease and ulcerative colitis (UC), is a chronic non-specific inflammatory disease of the gastrointestinal tract (1). IBD’s worldwide prevalence has become a global public health problem, but its pathogenesis is still unclear (2). Studies have suggested that the main etiology of IBD is an abnormal immune response to complex environmental factors in genetically susceptible individuals (3). This situation ultimately manifests as an abnormal immune system response, abnormal cytokine production, intestinal flora dysbiosis, and impaired intestinal mucosal barrier function. One of the key factors that clinicians find difficult in treating UC is disruption of the intestinal epithelial barrier (3–6). Current treatments for IBD include medical management (rest and nutritional support), pharmacological therapy (anti-inflammatory drugs and immunomodulators), and surgical treatment (total colectomy) (3, 7). An in-depth understanding of the molecular mechanisms underlying the pathogenesis of UC is necessary to enable the development of new strategies and approaches for its treatment.

The circadian clock is a highly conserved endogenous molecular system that controls circadian rhythms on a 24-hour cycle to ensure that internal physiology is synchronized with external environmental changes (8). The main circadian pacemaker in mammals is located in the suprachiasmatic nucleus (SCN) of the hypothalamus. This pacemaker transmits signals from the external environment, such as light and dark cycles, feeding behavior, and locomotion, to the periphery via endocrine and neural connectivity pathways. This process in turn translates from changes in the expression of biological clock genes to changes in the expression of clock control genes, thereby directly or indirectly regulating many physiological functions (9). Biological clocks are present in most tissues and organs, and are closely related to growth, body temperature, hormone secretion, metabolism, and the immune response. In particular, biological clocks are related to many physiological functions of the colon, such as barrier function represented by intestinal permeability, and also exhibit day-night rhythms (10–12). However, because of shift work, jet lag, and nighttime eating, circadian rhythm disruption (CRD) is occurring in an increasing number of people in the current society,. which is associated with many diseases, such as cancer, metabolic syndrome, and cardiovascular disease (13–15). And the relationship between CRD and IBD has been confirmed by several studies:circadian rhythm gene expression patterns are disturbed in patients with IBD (16); disruption of the circadian clock by sleep disorders increases the risk of IBD and the severity of disease in patients with IBD (17, 18); mice with disrupted central circadian rhythms are more susceptible to dextran sodium sulfate (DSS) or 2,4,6-trinitro-benzenesulfonic acid injury and exhibit more severe colitis (19–21), accompanied by a decline in the richness and diversity of the gut microbiota (22); similarly, mice with disturbed peripheral rhythms also exhibit more severe colitis, which may be related to disruption of intestinal barrier function, reduced relative abundance of short-chain fatty acid (SCFA)-producing bacteria and elevated phosphorylated STAT3 (23). The aryl hydrocarbon receptor nuclear translocation-like protein 1 (Bmal1) is a central transcriptional activator of the mammalian biological clock that plays a crucial role in the life activities of the organism (24). A heterodimer composed of BMAL1 and CLOCK mediates the transcription of tissue-specific genes and other negative feedback loops by binding to the E-box element in the promoter. This process forms the molecular basis of circadian rhythms (25), and disruption or deletion of Bmal1 can cause a complete loss of circadian rhythms in the organism and consequently result in a series of physiological abnormalities (24). A loss of rhythmic oscillations in Breg cells in intestinal epithelial lymphocytes, a reduction in the number of Breg+ PDL1+ cells, and dysfunction of CD4+ T cells in mice lacking the core biological clock gene *Bmal1* promote DSS-induced colitis and IBD-related colorectal cancer in mice (26). Furthermore, the circadian rhythm of regeneration in Bmal1-/- mutant mice is disrupted and reduced during recovery from DSS-induced colitis, with a concomitant reduction in cell proliferation (27). The above-mentioned evidence suggests that Bmal1 is closely associated with normal physiological functions of the gut. Specifically, the circadian protein Bmal1 is a negative regulator of IBD; however, the intrinsic mechanism of how Bmal1 mediates IBD is unknown.

Intestinal barrier dysfunction is an important pathogenic mechanism of UC (3). The physical barrier established by tight junctions (TJs) between intestinal epithelial cells (IECs) protects the body’s intestinal mucosa from external factors, such as pathogenic microorganisms (28). Autophagy is a highly conserved process in eukaryotic evolution by which damaged organelles or malfunctioning cytoplasmic proteins are transported to lysosomes for degradation. As a protective mechanism, autophagy plays a crucial role in maintaining the stability of the intestinal lining, regulating intestinal microbes and the immune response. A relationship between autophagy and intestinal barrier function has been demonstrated. Autophagy maintains intestinal barrier function by attenuating intestinal cell death and regulating TJ proteins (29, 30). However, a lack of autophagy is associated with a variety of human diseases including IBD (31). Bmal1 interacts with autophagy to play a major role in the development of disease. HSA-let-7f-1–3p, which is a micro RNA targeting the circadian gene Bmal1, can mediate the process of intervertebral disc degeneration by regulating autophagy (32). Additionally, Bmal1 overexpression is effective against angiotensin II-induced cardiac hypertrophy by regulating autophagy (33). However, to date, no studies have associated Bmal1 with autophagy in colitis and intestinal barrier function.

Therefore, in this study, we first investigated the effects of CRD on UC and found that CRD significantly exacerbated colitis in mice by impairing intestinal barrier function and increasing apoptosis of IECs. Mechanistically, CRD resulted in the downregulation of Bmal1 expression, while Bmal1 regulated intestinal barrier function by regulating autophagy. Taken together, our study suggests a protective role of Bmal1 in IBD. These findings expand new perspectives for studying the molecular mechanisms of circadian rhythms in colitis.

## 2 Materials and methods

### 2.1 Ethical considerations

All mouse experiments were approved by the Laboratory Animal Ethics Committee of Tongji Hospital and conducted in accordance with relevant guidelines and regulations.

### 2.2 Chemicals and reagents

Fluorescein isothiocyanate (FITC)-dextran 4000 (FD-4), lipopolysaccharide (LPS), 3-methyladenine (3-MA), wortmannin, and rapamycin were purchased from Sigma-Aldrich (St. Louis, MO). Tumor necrosis factor (TNF)-α and interferon (IFN)-γ were obtained from PeproTech (Rocky Hill, NJ). Dulbecco’s modified Eagle’s medium and fetal bovine serum were procured from GIBCO (Carlsbad, CA). Negative control lentivirus (LV-Vector), Bmal1-expressing lentivirus (LV-Bmal1), negative control-shRNA lentivirus (LV-shNC), and Bmal1-shRNA lentivirus (LV-shBmal1) were purchased from GeneChem (Shanghai, China). Primary antibodies against Bmal1 (1:1000), Bax (1:1000), and cleaved caspase-3 (1:500) were purchased from Abcam (Cambridge, UK). Bcl-2 (1:1000) was purchased from Abclonal (Wuhan, China). ZO-1 (1:5000), Occludin (1:5000), Claudin-1 (1:1000), and β-actin (1:5000) were purchased from Proteintech (Wuhan, China). Antibodies against LC3B (1:1000), Beclin-1 (1:1000), and P62 (1:1000) were purchased from Cell Signaling Technology (Danvers, MA).

### 2.3 Cell culture

The Caco-2 cell line was purchased from the China Center for Type Culture Collection (Wuhan, China). These cells were routinely cultured in Dulbecco’s modified Eagle’s medium supplemented with 10% fetal bovine serum and 1% penicillin/streptomycin solution in a humidified atmosphere (95% air/5% CO_2_) at 37°C. The cultured medium was replaced every 2 days.

### 2.4 Cell transfection

Caco-2 cells were infected with lentivirus to overexpress Bmal1 (LV-Bmal1) or knockdown Bmal1 (LV-shBmal1). After 72 hours of infection, the cells were then screened for 2 weeks using 5 μg/ml puromycin. Selected cells were identified as stable cell lines and used for the next experiments. The shBmal1 sequence was GCAGAATGTCATAGGCAAGT.

To visualize autolysosomes/autophagosomes, mRFP-GFP-LC3 lentivirus was transfected into Caco-2 cells. After appropriate interventions, the cells were fixed with 4% paraformaldehyde and observed with a Nikon Eclipse Ti laser confocal microscope (Nikon, Tokyo, Japan) after being sealed. Autophagosomes were characterized by co-localized green fluorescent protein (GFP) and red fluorescent protein (RFP) fluorescence, whereas autolysosomes showed RFP dots without GFP.

### 2.5 Detection of Caco-2 monolayer integrity and permeability

The integrity and permeability of the Caco-2 monolayer were determined by estimating transepithelial electrical resistance (TEER) and FD-4 permeability as described previously (34, 35). Briefly, Caco-2 cells were seeded at a density of 1 × 10^5^/cm^2^ cells on 0.4-μm 12-well hanging cell culture inserts (Corning, NY) and cultured for 21 days, with fluid changes every 2 days for the first 7 days and daily for the last 14 days. When cells were raised to day 21, Transwell plates were slowly washed 3 times with Hank’s balanced salt solution (HBSS) pre-warmed to 37°C. TEER values were measured using an EMD Millipore Millicell-ERS2 Volt-Ohm Meter (Millipore). The entire process was carried out at a 37°C constant temperature to guarantee the stability and accuracy of the value. Three different areas were selected for measuring the TEER value of each well. The final TEER value for each well was the average of the measurements for each well minus the blank control value multiplied by the area of each well of the Transwell plate. To measure the permeability of Caco-2 monolayers, monolayers pretreated with or without 10 ng/ml TNF-α/IFN-γ for 6 hours were washed twice with HBSS, and then 200 μL of FD-4 solution (1 mg/mL in HBSS) was added. After 2 hours of incubation, the concentration of FD-4 in samples extracted from the substrate chamber was evaluated using a fluorescent 96-well plate reader (Biotek, Vermont) at an excitation wavelength of 480 nm and an emission wavelength of 525 nm.

### 2.6 Mouse models

To investigate the effect of CRD on colitis, 120 male C57BL/6 mice were randomly divided into 4 groups: (a) wild type (WT) group; (b) DSS: WT group; (c) CRD group; and (d) DSS: CRD group. WT group mice were kept in a normal light-dark cycle environment (12 hours light, 12 hours dark) and CRD group mice were kept under constant light for 4 weeks (36). A concentration of 3% DSS (MP Biomedicals, Solon, OH) was added to the drinking water of mice for 7 days to construct the colitis model according to previous studies (37). In the DSS: CRD group, mice were kept under constant light for four weeks and 3% DSS was added to the drinking water at the beginning of the fourth week for seven days. Finally, all mice were sacrificed at the end of the fourth week.

To investigate the association between Bmal1 and autophagy and their role in colitis *in vivo*, we used Bmal1 knockout male C57BL/6 mice provided by Shulaibao Biotechnology (Wuhan, China). The strain information for Bmal1-/- mice is provided at https://www.jax.org/strain/009100. We divided the mice into 4 groups: (a) WT group; (b) DSS: WT group; (c) DSS: Bmal1-/- group, and (d) DSS: Bmal1-/- + rapamycin group (2 mg/kg/d, intragastric administration for 7 days), each group consisted of six mice.

The mice colitis model was modeled as described above. All mice were sacrificed at the end of the seventh day. Mice were weighed daily and checked for blood in the feces.

### 2.7 Disease activity index score

The daily observation indices during mouse colitis modeling mainly included changes in body weight, fecal properties, and blood in the feces. The disease activity index (DAI) was calculated as follows: DAI = (weight loss score + fecal trait score + blood in the feces)/3. The scale of the DAI is as follows: 0 points indicates no weight loss, normal fecal traits, and no blood in the feces; 1 point indicates weight loss of 1% to 5%, loose stools, and stool occult blood; 2 points indicate weight loss of 5% to 10%, loose stools, and stool occult blood; 3 points indicate weight loss of 10% to 15%, loose stools, and bloody stools; and 4 points indicate weight loss of ≥ 15%, loose stools, and bloody stools.

### 2.8 Histological analysis

A histological evaluation and scoring of mouse colon tissue samples were performed according to criteria that have been previously determined (38).

### 2.9 Intestinal permeability assay

The intestinal permeability of mice was measured according to a published protocol (34).

### 2.10 RNA isolation and quantitative reverse transcription-polymerase chain reaction

RNA from cells or colon tissues was isolated using the FastPure Cell/Tissue Total RNA Isolation Kit and converted to cDNA using the HiScript II First Strand cDNA Synthesis Kit according to standard protocols. ChamQ Universal SYBR qPCR Master Mix was used for quantitative reverse transcription-polymerase chain reaction (qRT-PCR) amplification. The above-mentioned reagents were supplied by Vazyme (Nanjing, China). The qRT-PCR primers used in this study were synthesized and provided by Tsingke Biotechnology (Beijing, China), and the sequences are shown in **Table 1**.

**Table 1:** Primer sequences used in the study.

Primer name	Primer sequences
**Human-Bmal1 sense:**	5′-AAGGGAAGCTCACAGTCAGAT-3′
**Human-Bmal1 antisense:**	5′-GGACATTGCGTTGCATGTTGG-3
**Human-Claudin-1 sense:**	5′-CCTCCTGGGAGTGATAGCAAT-3
**Human-Claudin-1 antisense:**	5′-GGCAACTAAAATAGCCAGACCT-3′
**Human-Occludin sense:**	5′-ACAAGCGGTTTTATCCAGAGTC-3′
**Human-Occludin antisense:**	5′-GTCATCCACAGGCGAAGTTAAT-3′
**Human-ZO-1 sense:**	5′-CAACATACAGTGACGCTTCACA-3′
**Human-ZO-1 antisense:**	5′-CACTATTGACGTTTCCCCACTC-3′
**Human-Actin sense:**	5′-CACTCTTCCAGCCTTCCTTC-3′
**Human-Actin antisense:**	5′-GTACAGGTCTTTGCGGATGT-3
**Mouse-Bmal1 sense:**	5′-ACAGTCAGATTGAAAAGAGGCG-3′
**Mouse-Bmal1 antisense:**	5′-GCCATCCTTAGCACGGTGAG-3′
**Mouse-Clock sense:**	5′-ATGGTGTTTACCGTAAGCTGTAG-3′
**Mouse-Clock antisense:**	5′-CTCGCGTTACCAGGAAGCAT-3′
**Mouse-Cry1 sense:**	5′-CACTGGTTCCGAAAGGGACTC-3′
**Mouse-Cry1 antisense:**	5′-CTGAAGCAAAAATCGCCACCT-3′
**Mouse-Cry2 sense:**	5′-CACTGGTTCCGCAAAGGACTA-3′
**Mouse-Cry2 antisense:**	5′-CCACGGGTCGAGGATGTAGA-3′
**Mouse-Per1 sense:**	5′-GAATTGGAGCATATCACATCCGA-3′
**Mouse-Per1 antisense:**	5′-CCCGAAACACATCCCGTTTG-3′
**Mouse-Per2 sense:**	5′-CTCCAGCGGAAACGAGAACTG-3′
**Mouse-Per2 antisense:**	5′-TTGGCAGACTGCTCACTACTG-3′
**Mouse-Actin sense:**	5′-CAGCCTTCCTTCTTGGGTATG-3′
**Mouse-Actin antisense:**	5′-GGCATAGAGGTCTTTACGGATG-3

### 2.11 Protein extraction and western blot

To extract cellular or mouse colon proteins after different treatment steps, samples were lysed in RIPA lysis buffer (Servicebio Technology, Wuhan, China) with a protease inhibitor cocktail (MedChemExpress, NJ). Protein concentrations were quantified using the BCA Protein Kit (Servicebio Technology). The supernatant of the lysate was mixed with the upwelling buffer (Servicebio Technology) and boiled at 100°C for 10 minutes. A total of 30 μg of total cellular protein or 50 μg of total tissue protein was separated by sodium dodecyl sulfate-polyacrylamide gel electrophoresis and then transferred to a polyvinylidene difluoride membrane (Millipore). The membrane was incubated overnight at 4°C in a configured specific primary antibody after being blocked with 5% skim milk for 1.5 hours. On the following day, the membrane was washed 3 times with tris-buffered saline Tween-20 for 10 minutes each time, followed by incubation with horseradish peroxidase-conjugated secondary antibody for 1.5 hour at room temperature. The membrane was then washed 3 times with tris-buffered saline Tween-20 for 10 minutes each time, and finally, protein bands were visualized using an ECL kit (NCM Biotech, Suzhou, China).

### 2.12 Immunohistochemistry

Mouse or human colon tissue was fixed in 4% paraformaldehyde, followed by paraffin embedding and sectioning. The prepared sections were used for subsequent immunohistochemical or immunofluorescence staining. To perform immunohistochemical staining, the sections were deparaffinized, hydrated, performed antigen retrieval, quenched by endogenous peroxidase, and blocked before dropwise addition of specific primary antibodies to the sections.

After overnight incubation of the specific primary antibody at 4°, IgG-HRP secondary antibody was added to the slide, and DAB was performed to observe the visible brown staining.

### 2.13 Immunofluorescence assays

Colon sections were processed in the same way as for immunohistochemistry. The secondary antibody used was CoraLite594-conjugated goat anti-rabbit IgG (H + L) antibody (Proteintech), and the nuclei were stained with 4’,6-diamidino-2-phenylindole (Servicebio Technology).

To perform cell immunofluorescence staining, the cells were fixed with 4% paraformaldehyde for 20 minutes at room temperature for Caco-2 cells or with methanol for 10 minutes at −20°C for Caco-2 monolayers. After permeabilization and blocking, the cells were incubated with specific primary antibodies overnight at 4°. The following day, the cells were incubated with CoraLite594-conjugated goat anti-rabbit IgG (H + L) secondary antibody for 1 hour in the dark. Subsequently, the nuclei were stained with 4’,6-diamidino-2-phenylindole, and the cells were observed by fluorescence microscopy.

### 2.14 Flow cytometry

The apoptosis rate of treated cells was assessed using flow cytometry. The Apoptosis Detection Kit (Yeasen, Shanghai, China) was used in this experiment. The cells were washed twice with phosphate-buffered saline before resuspension with a binding solution. This was followed by the addition of 5 μl of annexin V-FITC and 10 μl of propidium iodide (PI) to the cell mixture and incubation for 15 minutes at room temperature. Finally, the stained cells were evaluated by a FACScan flow cytometer (Becton Dickinson, San Diego, CA).

### 2.15 Terminal deoxynucleotidyl transferase-mediated dUTP nick-end labelling staining

Treated cells were stained to assess their apoptosis rate using the One-Step TUNEL Apoptosis Detection Kit (Yeasen) according to the manufacturer’s instructions.

### 2.16 Transmission electron microscopy

Transmission electron microscopy was used to visualize subcellular structures. Collected cells or colon tissue were fixed with 2.5% glutaraldehyde for 1 hour at room temperature, followed by fixation with osmium tetroxide and embedding in Spurr’s Epon. Ultra-thin sections (60 nm) were then cut and stained with uranyl acetate for 3 minutes. The sections were observed with a Hitachi 7500 electron microscope and photographed with a digital camera. The numbers of autophagosomes and autolysosomes were counted and plotted as histograms.

### 2.17 Statistical analysis

GraphPad Prism Software Version 8.0 (La Jolla, CA) was used to analyze the data. The data are shown as the mean ± standard deviation (SD). Significant differences between groups were analyzed by Student’s t-test, one-way analysis of variance (ANOVA) or two-way ANOVA, as appropriate. A value of p < 0.05 was considered statistically significant.

## 3 Results

### 3.1 CRD exacerbates DSS-induced acute colitis in mice

**Figure 1:**
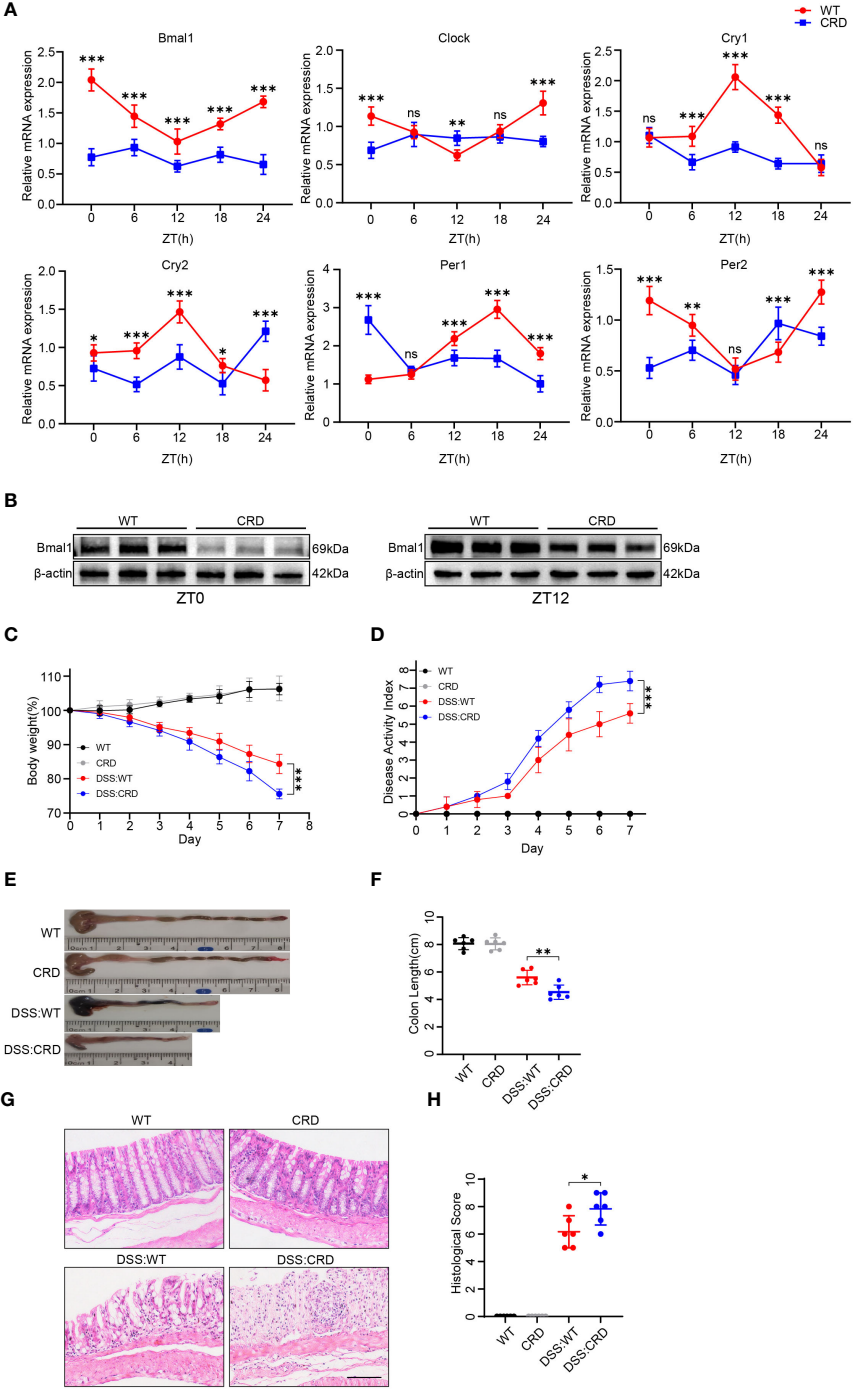
Circadian rhythm disruption aggravates DSS-induced colitis in mice. **(A)** qRT-PCR analysis of core clock genes in colon tissue of mice in the CRD group and in the WT group at different time points (6 mice/group). **(B)** Western blot analysis of the core clock gene Bmal1 in whole colon tissue samples from mice in the CRD and in the WT group at ZT0 and ZT12. **(C)** Body weight curve and **(D)** DAI of mice in each group (6 mice/group). **(E, F)** Colon length in each group. **(G, H)**, Representative hematoxylin and eosin–stained sections of distal colon tissue of mice and their corresponding histological score (6 mice/group). Scale bars: 100 μm. Data are presented as the mean ± SD. ns, means no significance, *p < 0.05, **p < 0.01, ***p < 0.001.

To study the effects of CRD on UC, we modeled CRD and CRD combined with acute colitis (DSS: CRD) by subjecting mice to constant light environment for 4 weeks and administering %3 DSS or no DSS for 7 days at the beginning of the fourth week. We found that mRNA expression levels of the core biological clock genes Bmal1, Clock, Per1, Per2, Cry1, and Cry2 in mouse colon tissue oscillated in a 24-hour cycle in the WT group, indicating clock rhythmicity. However, rhythmicity was disrupted in the colon tissue of mice raised in a constant light environment, which indicated successful construction of the CRD model (**Figure 1A**). Among the six biological clock genes in the CRD group compared with the WT group, Bmal1 mRNA expression was the only one that was significantly decreased at all time points. Western blot showed that Bmal1 protein levels in the CRD group were significantly lower than those in the WT group at zeitgeber time (ZT) 0 and ZT12 (**Figure 1B**, **Supplementary Figure 1A**), which is consistent with the findings of a previous study (39). We then examined the effect of CRD on acute colitis. We found that CRD significantly exacerbated DSS-induced acute colitis, as shown by a more pronounced weight loss (**Figure 1C**), a higher disease activity index (**Figure 1D**), shortened colon length (**Figures 1E, F**), marked disruption of epithelial crypt structure, and more immune cell infiltration (**Figures 1G, H**). However, CRD alone in the absence of colitis did not significantly affect the appearance or histological structure of the mouse colon (**Figures 1E–H**).

### 3.2 CRD exacerbates IEC apoptosis and impairs intestinal barrier function in the DSS-induced acute colitis model

**Figure 2:**
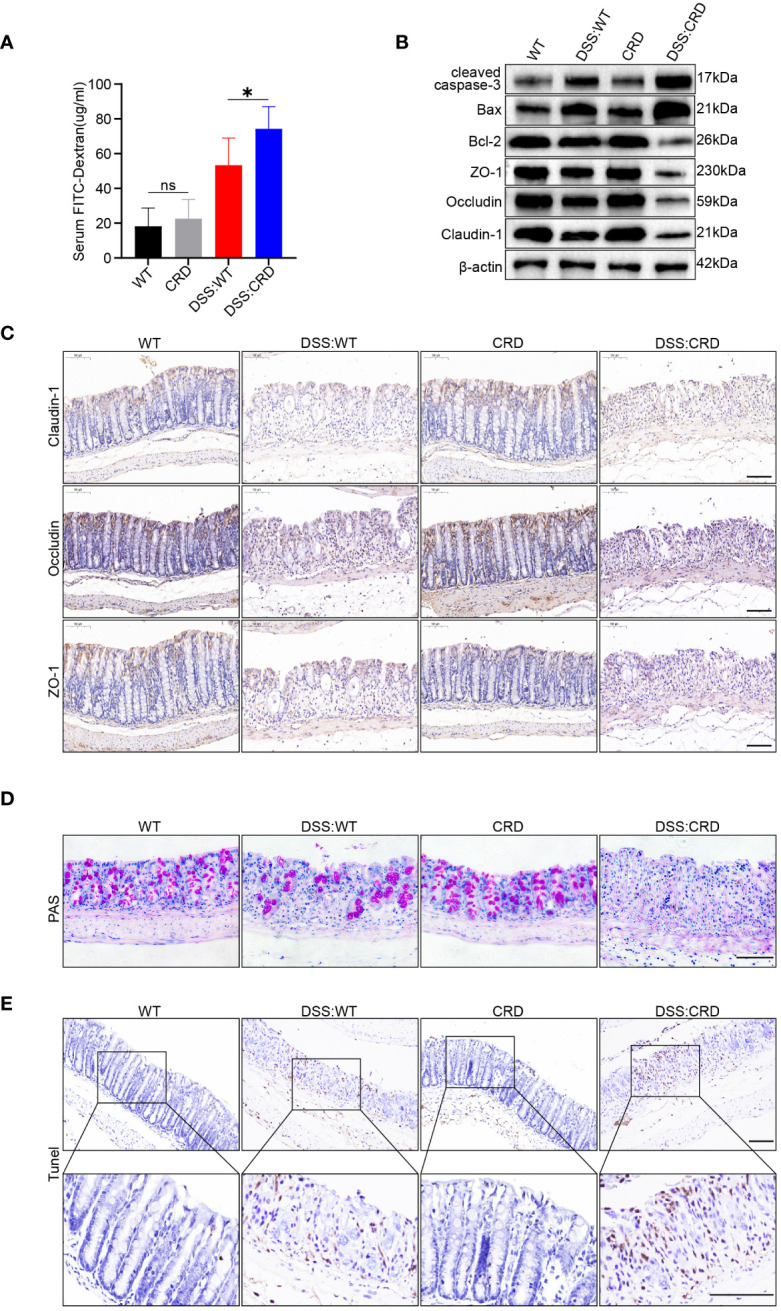
Circadian rhythm disruption impairs intestinal barrier function and promotes intestinal epithelial cell apoptosis in mice with DSS-induced colitis. **(A)** Intestinal permeability was measured by detecting blood serum FITC-dextran concentrations in different groups of mice (5 mice/group). **(B)** Western blot analysis of apoptosis-related proteins (cleaved caspase-3, Bax, and Bcl-2) and TJ proteins (Claudin-1, Occludin, and ZO-1) in whole colon tissue samples from each group of mice. **(C)** Immunohistochemical staining of Claudin-1, Occludin, and ZO-1 in colon sections from each group of mice. Scale bars: 100 μm. **(D)** Representative images of PAS staining of colon sections from each group of mice. Scale bars: 100 μm. **(E)** Representative images of TUNEL staining of colon sections from each group of mice. Scale bars: 100 μm. Data are presented as the mean ± SD. ns means no significance, *p < 0.05.

Previous studies have shown that the genes encoding the TJ proteins Claudin-1 and Occludin are expressed in a daily rhythm in WT mice (12). Additionally, excessive IECs apoptosis and intestinal barrier dysfunction are key factors in the pathogenesis of UC (40). We found that CRD did not significantly affect IEC apoptosis and intestinal barrier integrity in mice that were not subjected to DSS treatment. This finding was confirmed by the similar intestinal permeability (**Figure 2A**), apoptosis-related proteins (cleaved caspase-3, Bax and Bcl-2), TJ protein levels (**Figures 2B, C**, **Supplementary Figures 2A, B**), Periodic Acid-Schiff (PAS) staining (**Figure 2D**) and Terminal deoxynucleotidyl transferase-mediated dUTP nick-end labelling (TUNEL) staining in WT group and CRD group mice (**Figure 2E** and **Supplementary Figure 2C**). However, DSS: CRD group mice showed significantly higher intestinal permeability (**Figure 2A**), decreased TJ protein levels (**Figures 2B, C**, **Supplementary Figure 2A, B**), and more apoptotic IECs (**Figures 2B, E** and **Supplementary Figure 2C**) compared with DSS: WT group mice. DSS: CRD group mice also had fewer Goblet cells (**Figure 2D**), which protect the intestinal epithelial barrier function by secreting mucus (41). These results suggested that CRD aggravated impairment of intestinal barrier function and the apoptosis rate of IECs in the DSS-induced colitis model.

### 3.3 Bmal1 expression is decreased in DSS-induced mice model of colitis

**Figure 3:**
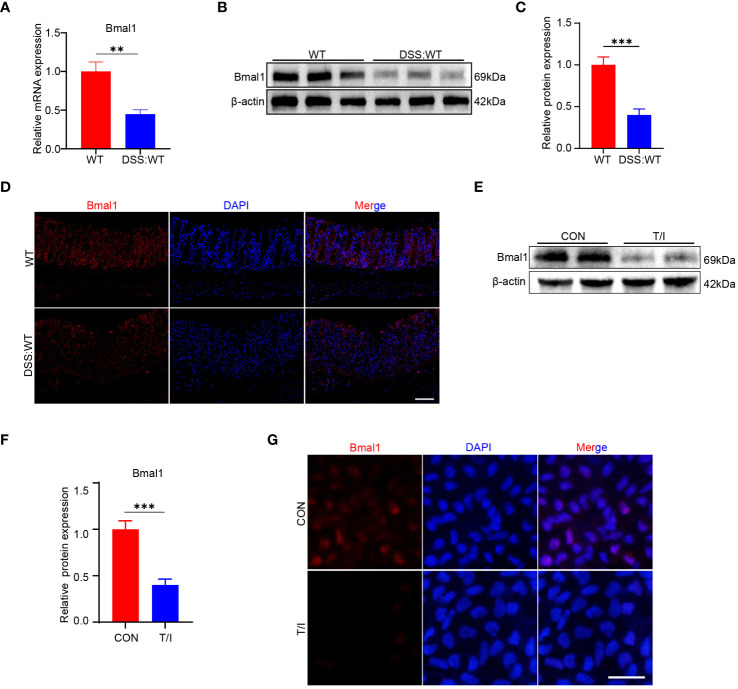
Bmal1 is decreased in mice with DSS-induced colitis and an *in vitro* model of inflammation. **(A–D)** qRT-PCR, western blot and its quantitative analysis, and immunofluorescent staining analysis of Bmal1 in whole colon tissue samples or colon sections from each group of mice (5 mice/group). Scale bars: 100 μm. **(E–G)**, Western blot and immunofluorescent staining analysis of Bmal1 in Caco-2 cells incubated with or without 10 ng/ml TNF-α and IFN-γ (T/I) for 12 hours. Scale bars: 50 μm. Data are presented as the mean ± SD. **p < 0.01, ***p < 0.001.

As mentioned above, Bmal1 mRNA expression was reduced in the CRD group at all 5 time points, and Bmal1 protein levels were reduced at ZT0 and ZT12 compared with the WT group (**Figures 1A, B** and **Supplementary Figure 1A**). Furthermore, previous studies have shown that Bmal1 is the central transcriptional activator of the mammalian biological clock, and its disruption or absence leads to complete loss of circadian rhythms in organisms (24). Therefore, we hypothesized that decreased Bmal1 expression plays a major role in CRD affecting the pathogenesis of UC. Our qRT-PCR, Western blot and immunofluorescence staining results showed that Bmal1 mRNA and protein levels of colon tissue in the DSS group were significantly lower than those in the WT group (**Figures 3A–D** and **Supplementary Figure 3A**). All colon tissues used for intercomparison were collected at the same time. Furthermore, our *in vitro* experiments showed that Bmal1 expression in Caco-2 cells was decreased after 12 hours of incubation with 10 ng/ml TNF-α/IFN-γ (**Figures 3E–G**). These results suggested that reduced Bmal1 expression may be involved in the pathogenesis of UC.

### 3.4 Bmal1 regulates IEC barrier function by modulating TJ protein levels and IEC apoptosis

**Figure 4:**
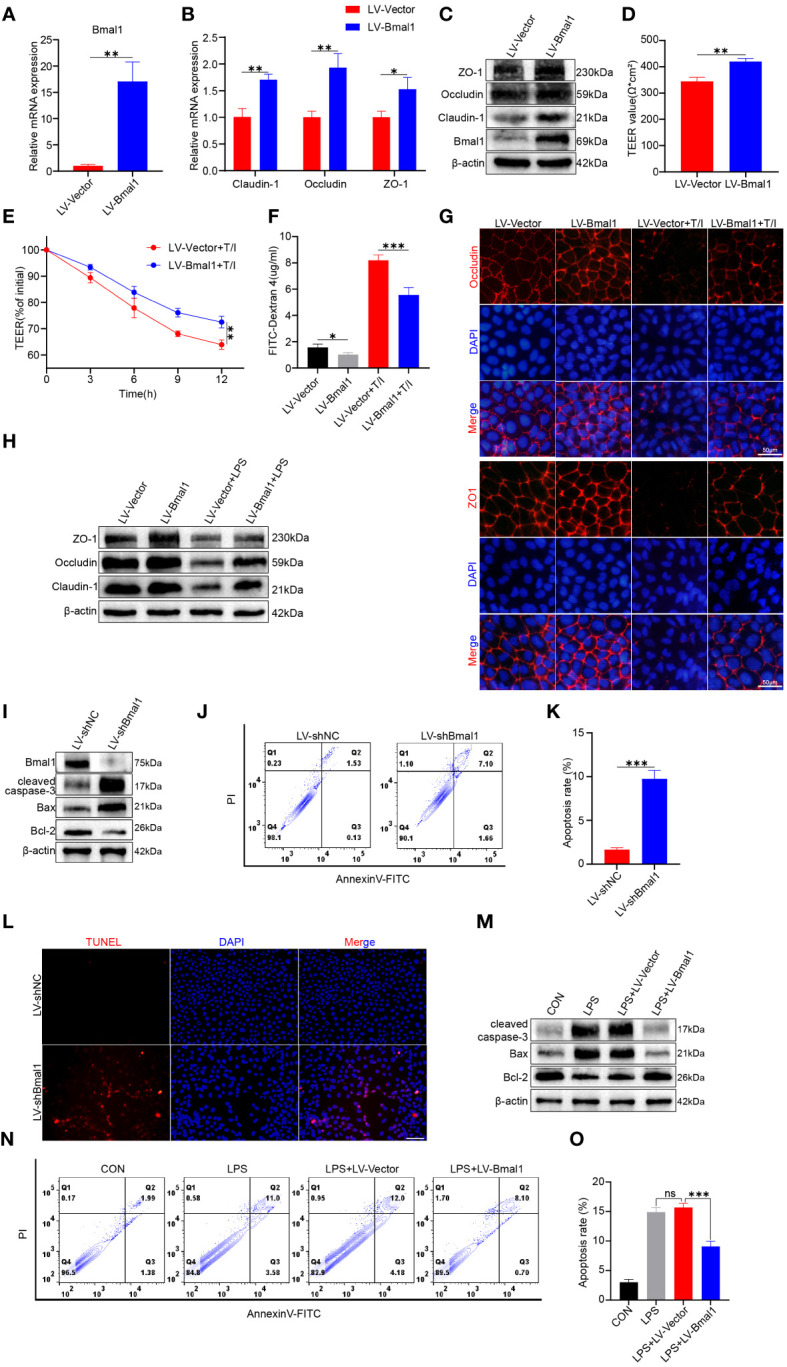
Bmal1 overexpression strengthens the epithelial barrier function of Caco-2 cell monolayers by promoting TJ protein expression and ameliorating apoptosis. Caco-2 cells were transfected with lentiviral vectors expressing Bmal1 or control lentiviral vectors, and then **(A)** qRT-PCR was used to assess Bmal1 gene expression. **(B, C)** qRT-PCR and western blot were used to assess Bmal1, Claudin-1, Occludin and ZO-1 gene and protein levels in Caco-2 cell monolayers. **(D)** TEER values of Caco-2 monolayers transfected with Bmal1 expressing lentiviral vectors or control lentiviral vectors. **(E)** TEER curves of Caco-2 monolayers transfected with Bmal1-expressing lentiviral vectors or control lentiviral vectors in the presence of 10 ng/mL TNF-α/IFN-γ(T/I). **(F)** After incubating Caco-2 monolayers with or without 10 ng/mL TNF-α/IFN-γ for 6 hours, FITC-dextran fluxes were measured in each group. **(G)** Representative images of immunofluorescent staining of ZO-1 and Occludin in Caco-2 monolayers incubated with or without 10 ng/mL TNF-α/IFN-γ for 12 hours. Scale bars: 50 μm. **(H)** Western blot analysis of Claudin-1, Occludin, and ZO-1 protein levels in Caco-2 cells incubated with or without 100 µg/ml LPS for 12h. **(I)** Caco-2 cells were transfected with shRNA lentivirus for Bmal1 (LV-shBmal1). Protein levels of Bmal1, cleaved caspase-3, Bax, and Bcl-2 were determined by western blot. **(J, K)** LV-shBmal1 and LV-shNC cells dual-stained with annexin V-FITC/PI were examined by flow cytometry and representative dot plots are shown. **(L)** Apoptosis of LV-shBmal1 and LV-shNC cells was assessed using the TUNEL assay. **(M)** The CON, LV-Bmal1, and LV-Vector groups were treated with or without 100 µg/ml LPS for 12h. Protein levels of cleaved caspase-3, Bax, and Bcl-2 in each group were analyzed by western blot. **(N, O)** Representative dot plots of each group of cells dual-stained with annexin V-FITC/PI with detection by flow cytometry. Data are presented as the mean ± SD. ns means no significance, *p < 0.05, **p < 0.01, ***p < 0.001.

As shown above, mice in the CRD group had decreased Bmal1 expression, and aggravated impairment of intestinal epithelial barrier function and increased apoptosis were observed in DSS: CRD group compared with DSS: WT group. To further investigate the potential regulatory effects of Bmal1 on intestinal epithelial barrier function, we transfected Caco-2 cells using LV-Bmal1 and LV-Vector, and verified Bmal1 mRNA expression by qRT-PCR (**Figure 4A**) and western blot (**Figure 4C**). In addition, mRNA and protein levels of Claudin-1, Occludin and ZO-1 were higher in the LV-Bmal1 group than in the LV-Vector group (**Figures 4B, C** and **Supplementary Figure 4A**). Measurements of TEER values in Caco-2 monolayers showed that Bmal1 overexpression enhanced the barrier function of Caco-2 monolayers (**Figure 4D**). Additionally, TEER values of Caco-2 monolayers expressing high Bmal1 levels declined to a lesser extent after the administration of TNF-α/IFN-γ (**Figure 4E**). This finding suggested that Bmal1 overexpression attenuated TNF-α/IFN-γ-induced impairment of the monolayer membrane barrier function in Caco-2 cells. FITC-dextran flux experiments also showed that FITC-dextran was less likely to pass through monolayers in the LV-Bmal1 group than in the LV-Vector group, regardless of the presence of TNF-α/IFN-γ (**Figure 4F**). Immunofluorescence staining showed that overexpression of Bmal1 in Caco-2 monolayers prevented the decrease in expression and disruption of localization of TJ proteins induced by TNF-α/IFN-γ (**Figure 4G**). Western blot showed that Bmal1 overexpression alleviated LPS-induced decreased TJ protein expression in Caco-2 cells (**Figure 4H** and **Supplementary Figure 4C**). Previous studies have reported that excessive epithelial cell death can lead to disruption of the intestinal barrier (42). Therefore, we evaluated the effect of Bmal1 on epithelial cell apoptosis. We knocked down Bmal1 in Caco-2 cells by transfecting lentivirus. Western blot, flow cytometry, and TUNEL staining suggested that Bmal1 knockdown induced apoptosis in Caco-2 cells and elevated cleaved caspase-3 Bax protein levels but decreased Bcl-2 protein level. (**Figures 4I–L** and **Supplementary Figures 2B, D**). However, Bmal1 overexpression alleviated the LPS-induced apoptosis in Caco-2 cells (**Figures 4M–O** and **Supplementary Figures 4E, F**), which is an *in vitro* model for mimicking IEC epithelial barrier damage and apoptosis *in vivo* (43).

The above-mentioned results suggest that Bmal1 can regulate the barrier function of IECs by modulating the expression of TJ proteins and apoptosis of IECs.

### 3.5 Bmal1 regulates IEC barrier function by activating autophagy *in vitro*

**Figure 5:**
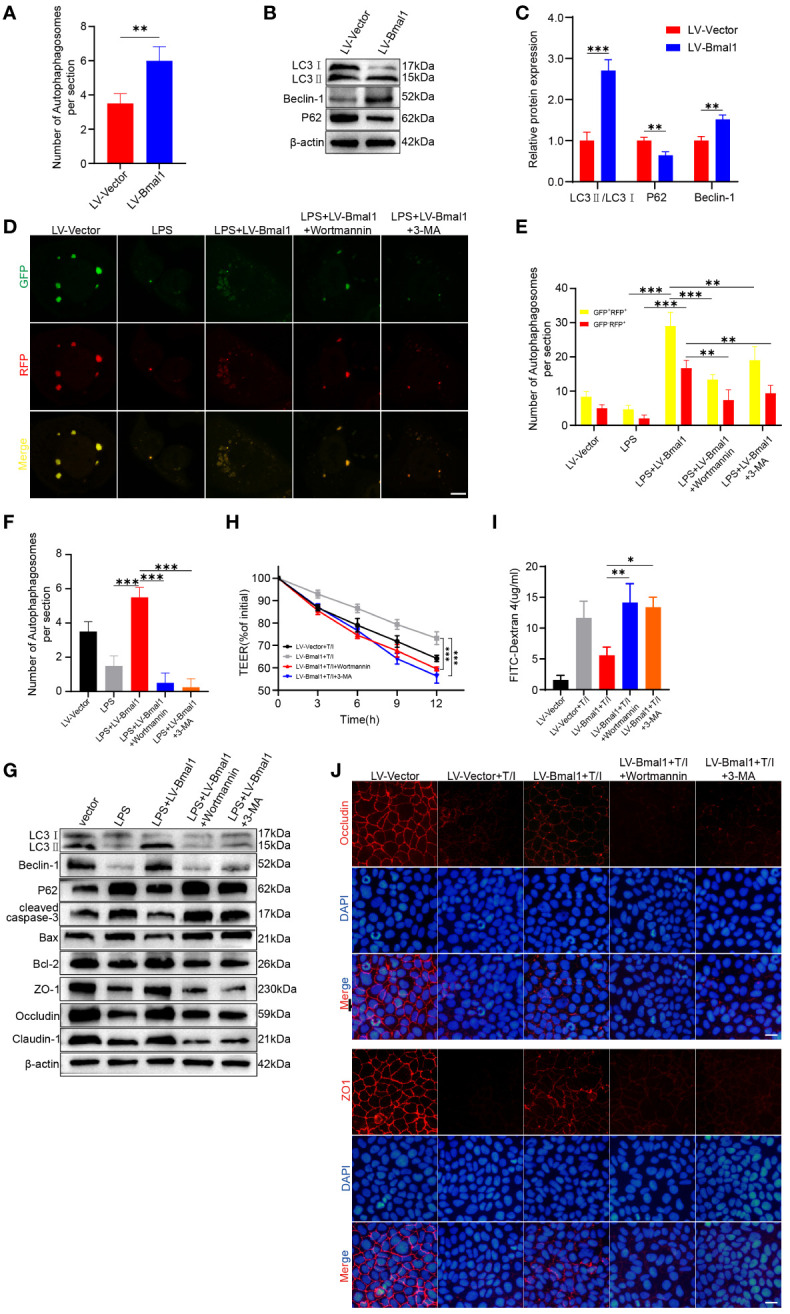
Bmal1 increases TJ protein levels and ameliorates apoptosis by regulating autophagy. **(A)** Quantitative analysis of the number of autophagosomes in each group. **(B)** Western blot analysis of LC3, Beclin-1, and P62 protein levels in the LV-Bmal1 and LV-Vector groups. **(C)** Quantitative analysis of the protein levels of LC3, Beclin-1, and P62 in the LV-Bmal1 and LV-Vector groups. **(D)** Representative fluorescent images representing autophagosomes (yellow puncta) and autolysosomes (red puncta), and **(E)** corresponding quantification of the number of intracellular autophagosomes and autolysosomes in Caco-2 cells transfected with Bmal1-expressing lentiviral vectors or control lentiviral vectors. The cells were treated with or without LPS (100 µg/ml) for 12h, pretreated with wortmannin (100 nM) for 12h or 3-MA (5 mM) for 12h or not. Scale bars: 10 µm. **(F)** Quantitative analysis of the number of autophagosomes in each group. **(G)** Western blot analysis shows protein levels of LC3, Beclin-1, P62, cleaved caspase-3, Bax, Bcl-2, ZO-1, Occludin, and Claudin-1 in each groups cells as described above. **(H)** TEER curves of Caco-2 monolayers transfected with Bmal1-expressing lentiviral vectors or control lentiviral vectors in the presence of 10 ng/mL TNF-α/IFN-γ, pretreated with wortmannin (100 nM) for 12h or 3-MA (5 mM) for 12h or not. **(I)** After incubating Caco-2 monolayers pretreated with wortmannin (100 nM) for 12h or 3-MA (5 mM) for 12h or not with or without 10 ng/mL TNF-α/IFN-γ for 6 hours, FITC-dextran fluxes were measured in each group. **(J)** Representative images of immunofluorescent staining of ZO-1 and Occludin in Caco-2 monolayers in the presence of 10 ng/mL TNF-α/IFN-γ, pretreated with wortmannin (100 nM) for 12h or 3-MA (5 mM) for 12h or not. Scale bars: 50 µm. Data are presented as the mean ± SD. **p < 0.01, ***p < 0.001.

Specific deletion of Bmal1 in astrocytes affects autophagy and protein degradation kinetics (44), and Bmal1 can mediate the process of intervertebral disc degeneration by regulating autophagy (32). In addition, autophagy maintains intestinal barrier function by reducing IEC death and regulating TJ proteins (29, 30). To determine whether Bmal1 regulates IEC barrier function by modulating autophagy, we used transmission electron microscopy to visualize subcellular structures. The number of autophagosomes was significantly higher in the LV-Bmal1 group than in the LV-Vector group (**Supplementary Figure 5A** and **Figure 5A**). Western blot showed that the LC3II:I ratio and Beclin-1 levels were higher in the LV-Bmal1 group than in the LV-Vector group. However, P62 protein levels were significantly lower in the LV-Bmal1 group than in the LV-Vector group (**Figures 5B, C**). The western blot findings were confirmed by immunofluorescence staining of LC3B and P62 in the LV-Bmal1 and LV-Vector groups (**Supplementary Figure 5B**). These results suggested that Bmal1 overexpression in Caco-2 cells activated autophagy. To investigate the effect of Bmal1 on autophagic flux in Caco-2 cells in the presence of LPS, we transfected the cells with the mRFP-GFP-LC3 lentiviral reporter gene. Yellow spots fused by red (mRFP) and green spots (GFP) indicated autophagic vesicles, while autophagic lysosomes were shown as red spots because the acidic environment quenched the fluorescence of GFP. We found that the numbers of autophagosomes and autolysosomes were significantly higher in the LPS + LV-Bmal1 group than in the LPS group. However, when the autophagy inhibitors 3-MA and wortmannin were added, autophagic vesicles and autophagic lysosomes were reduced (**Figures 5D, E**). These findings suggested that the activation of autophagy by Bmal1 was abolished, which was also confirmed by transmission electron microscopy (**Supplementary Figure 5C** and **Figure 5F**). Western blot showed that the LC3II:I ratio and Beclin-1 levels were decreased and P62 levels were increased by the administration of autophagy inhibitors, and lower levels of TJ proteins (Claudin-1, Occludin, and ZO-1) and higher levels of apoptosis-related proteins (cleaved caspase-3 and Bax) can also be observed in the wortmannin- or 3-MA-treated group than in the LPS + LV-Bmal1 group (**Figure 5G** and **Supplementary Figure 5D**). Flow cytometry showed that the inhibition of apoptosis by Bmal1 overexpression was partly abolished by autophagy inhibitors (**Supplementary Figure 5E**). Furthermore, we also observed that the protective effect of overexpression of Bmal1 on Caco-2 monolayers was partly deprived when TNF-α/IFN-γ stimulation was applied to Caco-2 monolayers pretreated with autophagy inhibitors wortmannin or 3-MA (**Figures 5H–J** and **Supplementary Figure 5D**). The above-mentioned results indicated that the protective effect of Bmal1 on the barrier function of Caco-2 cells was weakened by the administration of autophagy inhibitors exogenously. In summary, Bmal1 regulated the barrier function of IECs by activating autophagy.

### 3.6 Bmal1 knockout does not significantly affect the colonic function of mice under baseline conditions

We investigated the role of Bmal1 in UC using the Bmal1-/- mouse strain. Western blot analysis of proteins extracted from whole colon tissue showed that Bmal1 was ablated in colon tissue (**Supplementary Figure 6A**).Our results showed that Bmal1-/- and WT groups had similar colon lengths (**Supplementary Figures 6B, C**), and there was no significant difference in the histological structure or distribution of Goblet cells between the groups of mice. Additionally, the proliferative activities of the two groups were approximately the same (**Supplementary Figure 6D**). These results suggested that knockout of Bmal1 did not significantly affect mouse colonic function under baseline conditions.

### 3.7 Bmal1 deficiency impairs intestinal barrier function by impairing autophagy, leading to exacerbation of DSS-induced colitis *in vivo*

**Figure 6:**
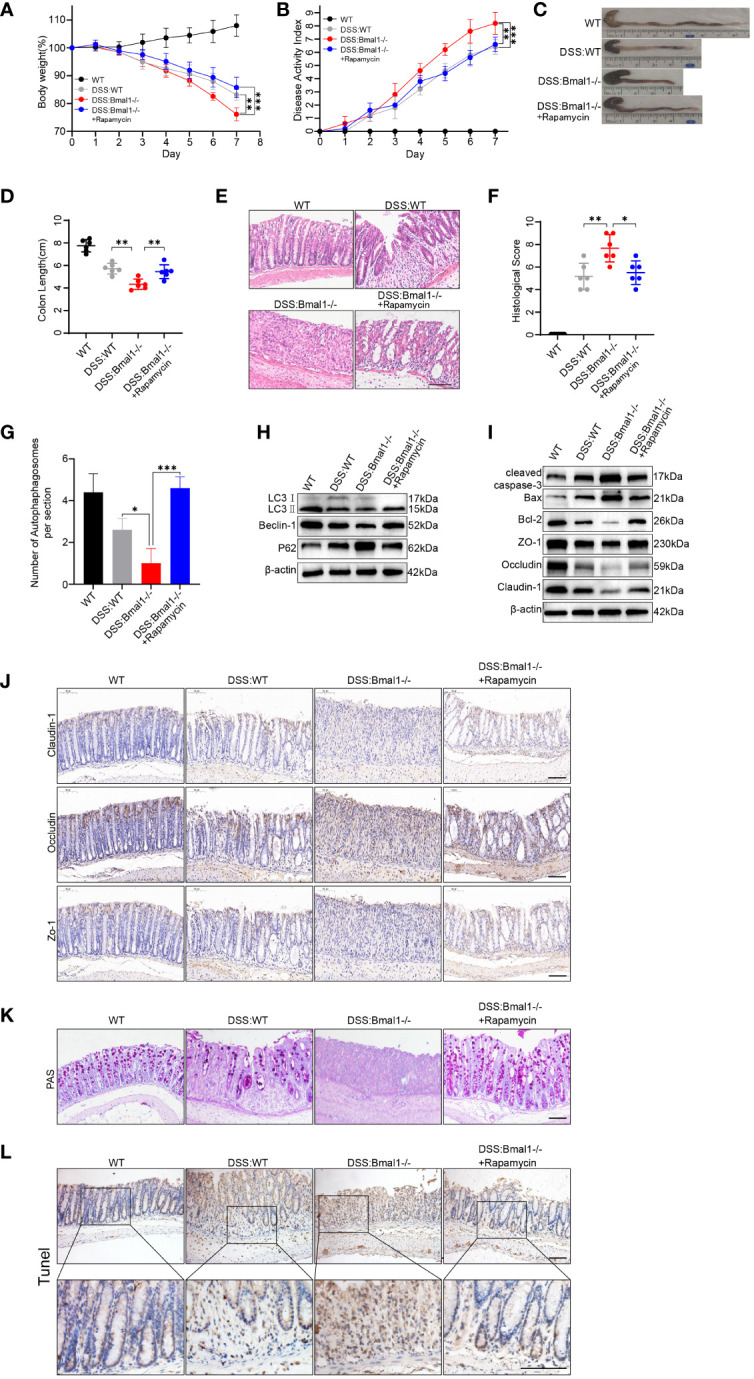
Bmal1 deficiency exacerbates DSS-induced colitis by inhibiting autophagy. **(A)** Body weight curve and **(B)** DAI of mice in each group (5 mice/group). **(C, D)** Length of the colon in each group of mice and its quantitative analysis (6 mice/group). **(E, F)** Representative hematoxylin and eosin staining images of distal colon tissue sections of each group of mice and their corresponding histological scores (6 mice/group). Scale bars: 100 μm. **(G)** Quantitative analysis of representative electron microscopy images of colon tissues from each group of mice (5 mice/group). **(H)** Western blot analysis of LC3, Beclin-1 and P62. **(I)** Western blot analysis of cleaved caspase-3, Bax, Bcl-2, Claudin-1, Occludin, and ZO-1 in whole colon tissue samples from each group of mice. **(J)** Immunohistochemical staining of Claudin-1, Occludin, and ZO-1 in colon sections from each group of mice. Scale bars: 100 μm. **(K)** Representative images of PAS staining of colon sections from each group of mice. Scale bars: 100 μm. **(L)** Representative images of TUNEL staining of colon sections from each group of mice. Scale bars: 100um. Data are presented as the mean ± SD. *p < 0.05, **p < 0.01, ***p < 0.001.

We assessed the severity of colitis and found that the DSS: Bmal1-/- group showed significantly greater weight loss (**Figure 6A**) and a higher DAI score (**Figure 6B**) than the DSS: WT group. The length of the colon was significantly shorter in the DSS: Bmal1-/- group than in the DSS: WT group (**Figures 6C, D**). Histopathological staining showed significant morphological differences in the colonic epithelium of DSS: Bmal1-/- group mice, as shown by more extensive epithelial detachment, ulceration, disruption of crypt structures, and thickening of muscular tissue than in the DSS: WT group. These findings resulted in significantly higher histological scores in DSS: Bmal1-/- group than in the DSS: WT group (**Figures 6E, F**). We also found that the use of the autophagy agonist rapamycin (DSS: Bmal1-/- + rapamycin group) alleviated colitis exacerbated by Bmal1 knockout (**Figures 6A–F**). We then further confirmed the role of the Bmal1–autophagy–gut barrier function axis in the pathogenesis of colitis. The DSS: Bmal1-/- group showed more severe impaired autophagy than the DSS: WT group, as shown by the results of transmission electron microscopy (**Supplementary Figure 7A** and **Figure 6G**), a decreased LC3II:I ratio and Beclin-1 protein levels, increased P62 protein levels (**Figure 6H** and **Supplementary Figure 7C**) and LC3B immunofluorescence staining of colon tissue sections (**Supplementary Figure 7B**). DSS: Bmal1-/- group mice had significantly higher intestinal permeability (**Supplementary Figure 7D**), lower TJ protein (Claudin-1, Occludin, and ZO-1) levels (**Figures 6I, J** and **Supplementary Figures 7E, F**), fewer Goblet cells (**Figure 6K**), higher protein levels of cleaved-caspase-3, Bax (**Figures 6I** and **Supplementary Figure 7E**) and more apoptotic IECs compared with DSS: WT group (**Figure 6L** and **Supplementary Figure 7G**). Furthermore, we also observed that all of the above-mentioned conditions were relieved with the use of rapamycin (DSS: Bmal1-/- + rapamycin group) (**Figures 6G–L** and **Supplementary Figures 7A–G**). In conclusion, we demonstrated that Bmal1 deficiency exacerbated DSS-induced colitis by impairing intestinal barrier function through impaired autophagy.

## 4 Discussion

UC is a circadian rhythms dysfunction-associated, most common chronic inflammatory bowel disease affecting the colon and rectum, and there is no effective cure for UC, so it is crucial to elucidate its pathogenesis and therapeutic targets (13, 17, 18, 45). Circadian rhythms play a crucial role in maintaining normal physiological functions of the body, and circadian disorders are thought to increase the risk of neurological, psychiatric, cardiometabolic, and immune disorder (46). The relationship between CRD and intestinal inflammation has been confirmed by numerous studies (19, 26, 27, 47). Altered expression of biological clock genes may exist as an early event in the pathogenesis of IBD (16). Our study found that Bmal1 expression was decreased in the colon of CRD mice, and downregulation of Bmal1 expression due to CRD or Bmal1 knockout lead to more severe colitis, as evidenced by more severe impairment of intestinal barrier function and more apoptosis IECs. *In vitro*, high expression of Bmal1 may protect Caco-2 cell monolayer membrane barrier function by increasing TJ proteins expression as well as inhibiting IECs apoptosis. Mechanistically, Bmal1 inhibits IECs apoptosis and increases TJ proteins expression by regulating autophagy, thus exerts a protective effect on the intestinal barrier function. This study reveals the mechanism by which CRD affects UC through downregulation of Bmal1 expression, providing a new target for UC treatment and drug exploitation.

Circadian rhythms present in mammals have the ability to regulate cellular and organ processes to adapt to the surrounding 24-hour light/dark cycle. The molecular basis of the biological clock consists of a series of clock genes, including Bmal1, the circadian motor output cycle kaput (Clock), cyclic genes (Per1, Per2, and Per3), and cryptochrome genes (Cry1 and Cry2) (48).Our study found that Bmal1 mRNA expression was the only one that was significantly decreased at all time points in the CRD mice model, given its centrality in the biological clock system, we chose it as the main object of our study. Furthermore, we observed decreased expression of Bmal1 in the colon of mice with DSS-induced colitis, which is consistent with previous studies (16, 39, 49), suggesting that Bmal1 may be a key factor in the process of CRD affecting UC pathogenesis.

Intestinal barrier dysfunction is a key factor influencing the pathogenesis of UC. The intestinal barrier restricts the free exchange of water, ions and macromolecules between the intestinal lumen and the intestinal epithelium as well as protects the body from intestinal microbial damage. TJ proteins including Claudins, Occludin and ZO-1 play an important role in regulating intestinal permeability (50). A previous study confirmed that Claudin-1 and Occludin show a circadian rhythm of expression in the mouse colon under the control of Bmal1-Clock (12). In addition, a recent study indicated that CRD alters intestinal barrier permeability, which is associated with dysregulated ß-catenin expression and altered TJ protein expression (39). According to our data, both CRD mice and Bmal1-/- mice exhibited more severe intestinal barrier dysfunction in the presence of DSS-induced colitis, which may be mediated by decreased TJ proteins (Claudin-1, Occludin and ZO-1) expression and increased apoptosis of IECs, thus aggravated the severity of colitis. Previous studies have found that Bmal1 regulated the expression of the TJ protein claudin-5, which was abundantly expressed at the blood-retinal barrier (iBRB) (51); Bmal1 deficiency lead to an increase in blood-brain barrier permeability (52). However, the role of Bmal1 in regulating intestinal epithelial cell function has not been well elucidated. Our gain-of-function studies results showed that overexpression of Bmal1 increased TJ proteins expression, inhibited the apoptosis of IECs under inflammation, decreased the permeability of the intestinal barrier, and protected the intestinal barrier function. Furthermore our loss-of-function studies showed that knockdown of Bmal1 induces apoptosis in intestinal epithelial cells, which is one of the key factors for the impaired intestinal barrier function. Thus, Bmal1 may serve as a potential target for repairing intestinal barrier function in UC patients.

**Figure 7:**
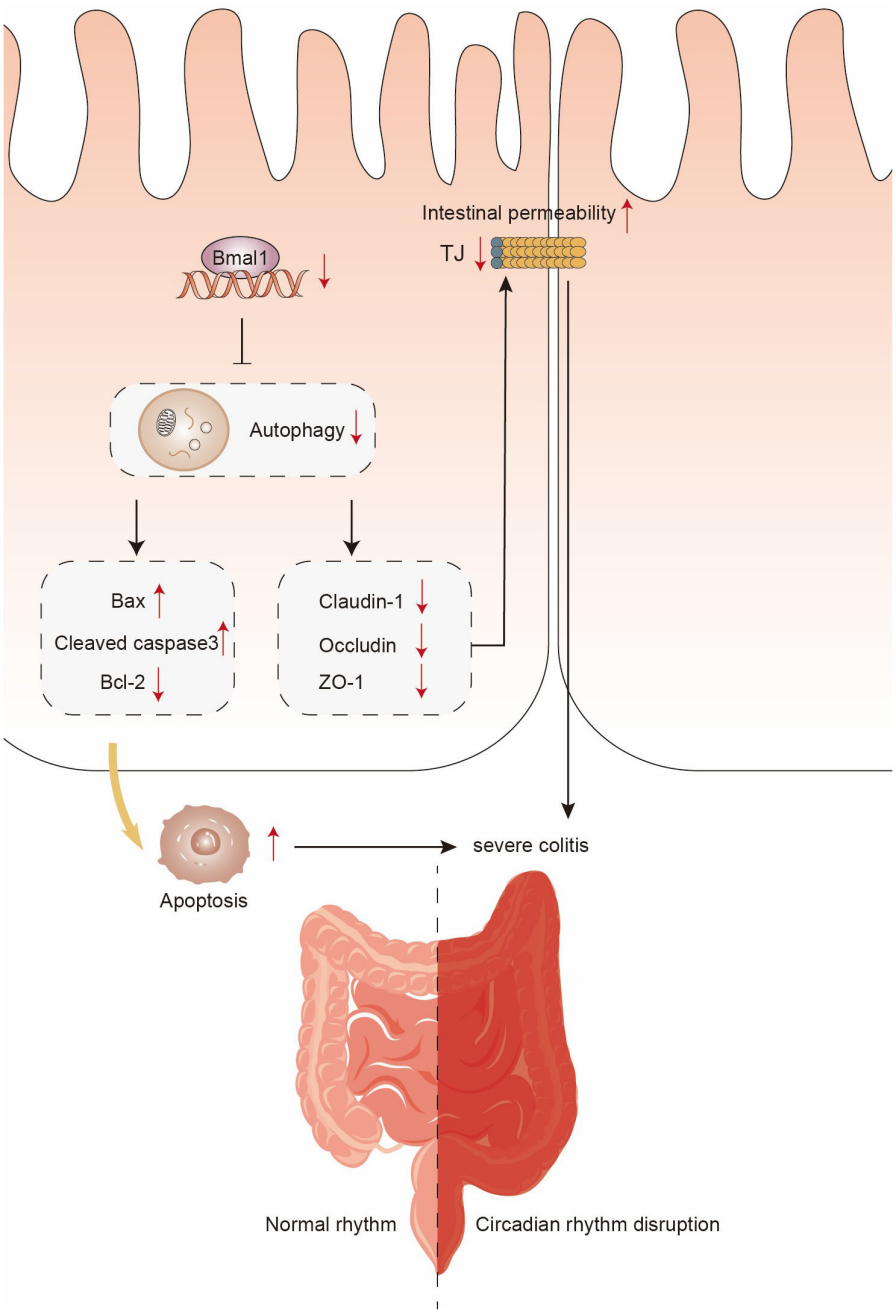
Schematic diagram of the possible mechanisms of CRD in the pathogenesis of UC.

Autophagy, a physiological process in which damaged organelles or malfunctioning cytoplasmic proteins can be translocated to lysosomes for degradation (29). A number of recent studies have reported that circadian rhythms and Bmal1 can modulate autophagy to influence physiological processes or disease progression: an intermittent time-restricted feeding (iTRF) dietary regimen may extend the healthy lifespan of flies by activating autophagy driven by circadian rhythms (53); autophagy in photoreceptors and retinal pigment epithelium is partly regulated by circadian rhythms (54); one of the important genes involved in the initiation of autophagy, autophagy-related 14 (Atg14), is regulated by circadian rhythms in mouse liver (55). Also, as a central component of the molecular basis of the biological clock, Bmal1 attenuates apoptosis and modulates extracellular matrix metabolism by activating autophagy in intervertebral disc degeneration (32). The role of autophagy in protecting intestinal barrier function by reducing intestinal cell death and regulating TJ proteins has been well established (29, 30). Our study found that Bmal1 inhibits IECs apoptosis and protects intestinal epithelial barrier function through activation of autophagy. Meanwhile, the use of the autophagy agonist rapamycin *in vivo* attenuates the more severe colitis caused by Bmal1 deficiency through inhibiting IECs apoptosis and enhancing intestinal epithelial barrier function. Based on our findings, we can make the inference that limited autophagy regulated by normal circadian rhythms protects intestinal barrier function, but dysregulation of autophagy caused by downregulation of Bmal1 expression due to CRD exacerbates the severity of UC (**Figure 7**).

However, the present study has some limitations. Firstly, no additional interventions such as increasing colonic Bmal1 expression or using drugs that modulate autophagy were performed in CRD mice to better confirm the CRD-Bmal1-autophagy axis in UC. Secondly, limited by experimental conditions, we used Bmal1 knockout mice rather than IECs -specific Bmal1 knockout mice in our *in vivo* study of the role of Bmal1 in epithelial cells in the pathogenesis of UC. In addition, more experiments are needed to further elucidate the specific mechanisms by which Bmal1 regulates autophagy.

## Data availability statement

The original contributions presented in the study are included in the article/**Supplementary Material**. Further inquiries can be directed to the corresponding authors.

## Ethics statement

The animal study was approved by Laboratory Animal Ethics Committee of Tongji Hospital. The study was conducted in accordance with the local legislation and institutional requirements.

## Author contributions

ZZ: Conceptualization, Data curation, Investigation, Methodology, Project administration, Writing – original draft. WL: Investigation, Project administration, Software, Visualization, Writing – original draft. XH: Data curation, Formal analysis, Investigation, Writing – review & editing. DT: Supervision, Validation, Writing – review & editing. WY: Methodology, Supervision, Validation, Writing – review & editing. ML: Supervision, Writing – review & editing. LC: Funding acquisition, Supervision, Writing – review & editing.
