# Supplementary material for: Circadian rhythm disruption-mediated downregulation of Bmal1 exacerbates DSS-induced colitis by impairing intestinal barrier
Source: Front Immunol. 2024 Jun 4;15:1402395. doi: 10.3389/fimmu.2024.1402395 (PMC11183104; doi:10.3389/fimmu.2024.1402395)
Supplement: Supplementary file 1 [file DataSheet_1.docx]

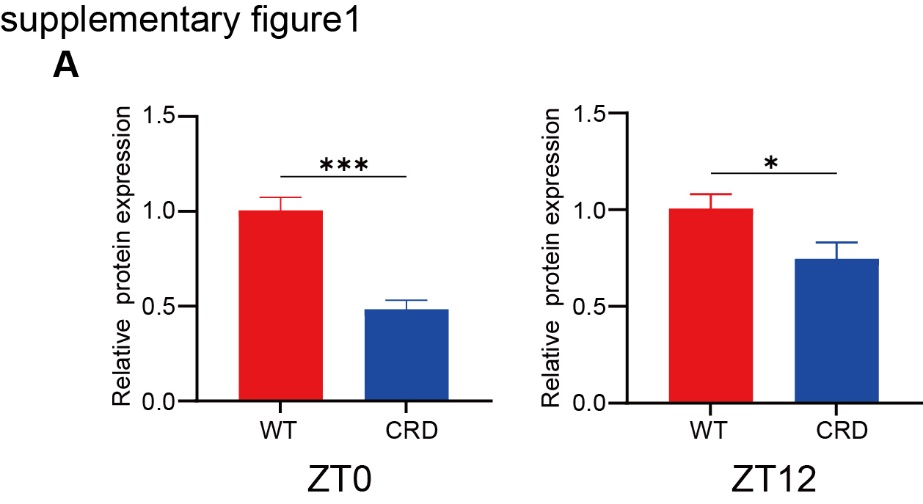


**Supplementary Fig. 1 Circadian rhythm disruption induced down-regulation of Bmal1**

**A** Quantitative analysis of the protein levels of Bmal1 in the WT and CRD groups. *p < 0.05, ***p < 0.001.


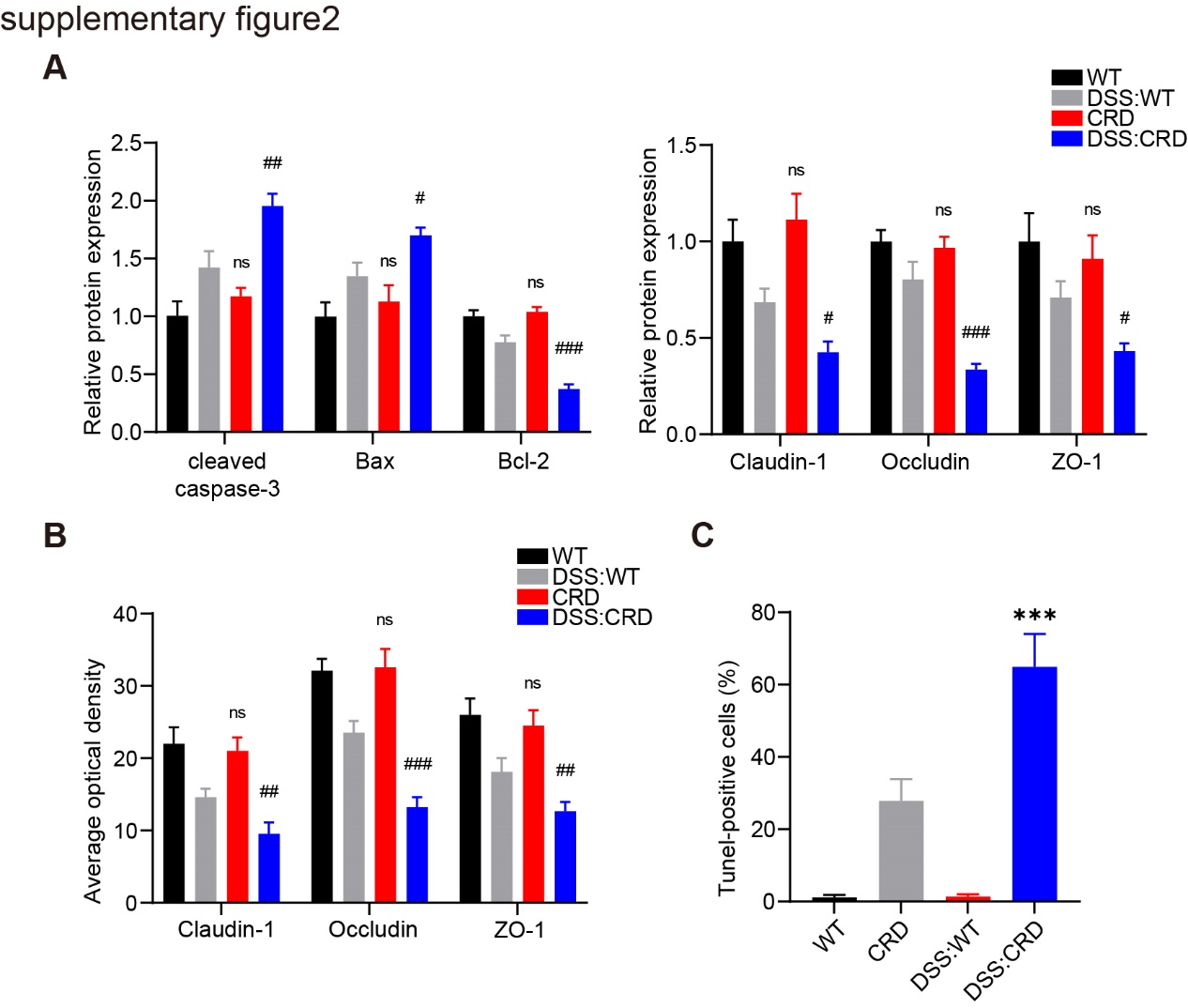


**Supplementary Fig. 2 CRD exacerbates IEC apoptosis and down-regulation of tight junction protein expression in the DSS-induced acute colitis model**

**A** Quantitative analysis of the protein levels of apoptosis-related proteins (cleaved caspase-3, Bax, and Bcl-2) and TJ proteins (Claudin-1, Occludin, and ZO-1) in each group. ns means no significance vs WT group,  ^#^ p < 0.05, ^##^ p < 0.01, ^###^ p < 0.001 vs DSS:WT group.

**B** Quantitative analysis of the immunohistochemical staining results of each group. ns means no significance vs WT group,  ^##^ p < 0.01, ^###^ p < 0.001 vs DSS:WT group.

**C** Quantitative analysis of the TUNEL staining results of each group. ***p < 0.001.


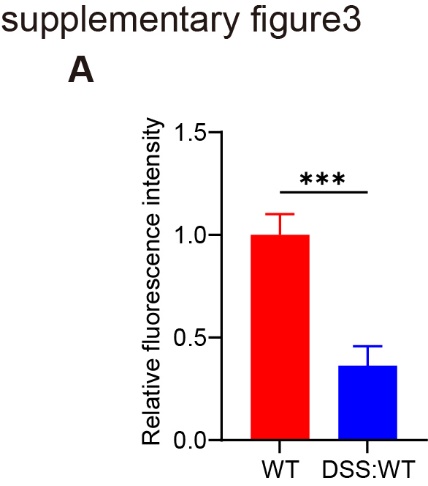


**Supplementary Fig. 3 Bmal1 is decreased in DSS-induced colitis.**

**A** Quantitative analysis of the immunofluorescence staining results of the WT and DSS:WT groups. ***p < 0.001.


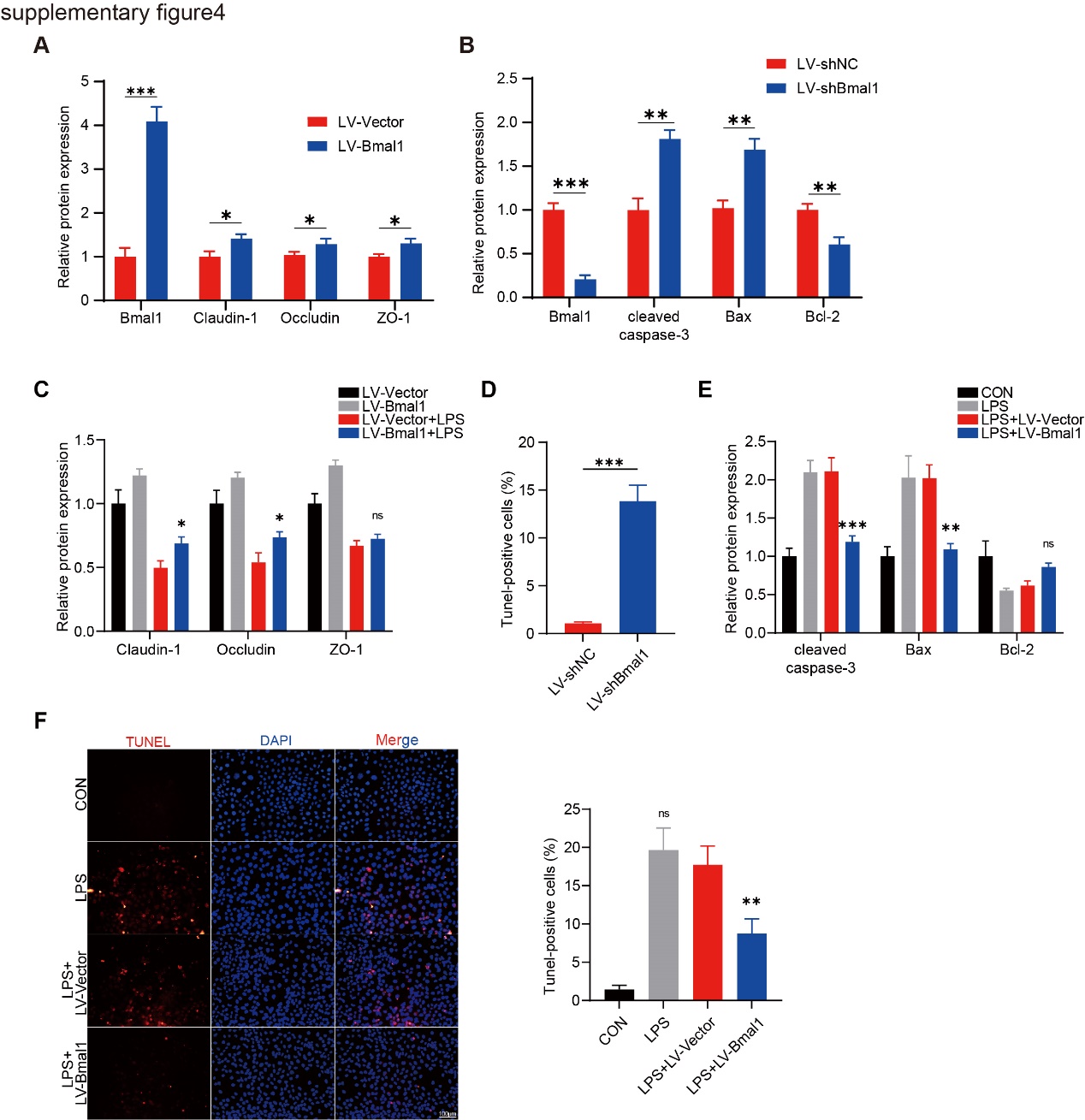


**Supplementary Fig. 4 Bmal1 overexpression strengthened the epithelial barrier function of Caco-2 cell monolayers**

**A** Quantitative analysis of the protein levels of Bmal1, Claudin-1, Occludin, and ZO-1 in the LV-Vector and LV-Bmal1 groups. *p < 0.05, ***p < 0.001.

**B** Quantitative analysis of the protein levels of Bmal1, cleaved caspase-3, Bax, and Bcl-2 in the LV-shNC and LV-shBmal1 groups. *p < 0.05, **p < 0.01, ***p < 0.001.

**C** Quantitative analysis of the protein levels of Claudin-1, Occludin, and ZO-1 in each group. ns means no significance, * p < 0.05 vs LV-Vector + LPS group.

**D** Quantitative analysis of the TUNEL staining results of the LV-shNC and LV-shBmal1 groups. ***p < 0.001.

**E** Quantitative analysis of the protein levels of cleaved caspase-3, Bax, and Bcl-2 in each group. ns : no significance, **p < 0.01, ***p < 0.001 vs LPS + LV-Vector group.

**F** Apoptosis of each group of cells was assessed using the TUNEL assay. ns : no significance, **p < 0.01 vs LPS + LV-Vector group.


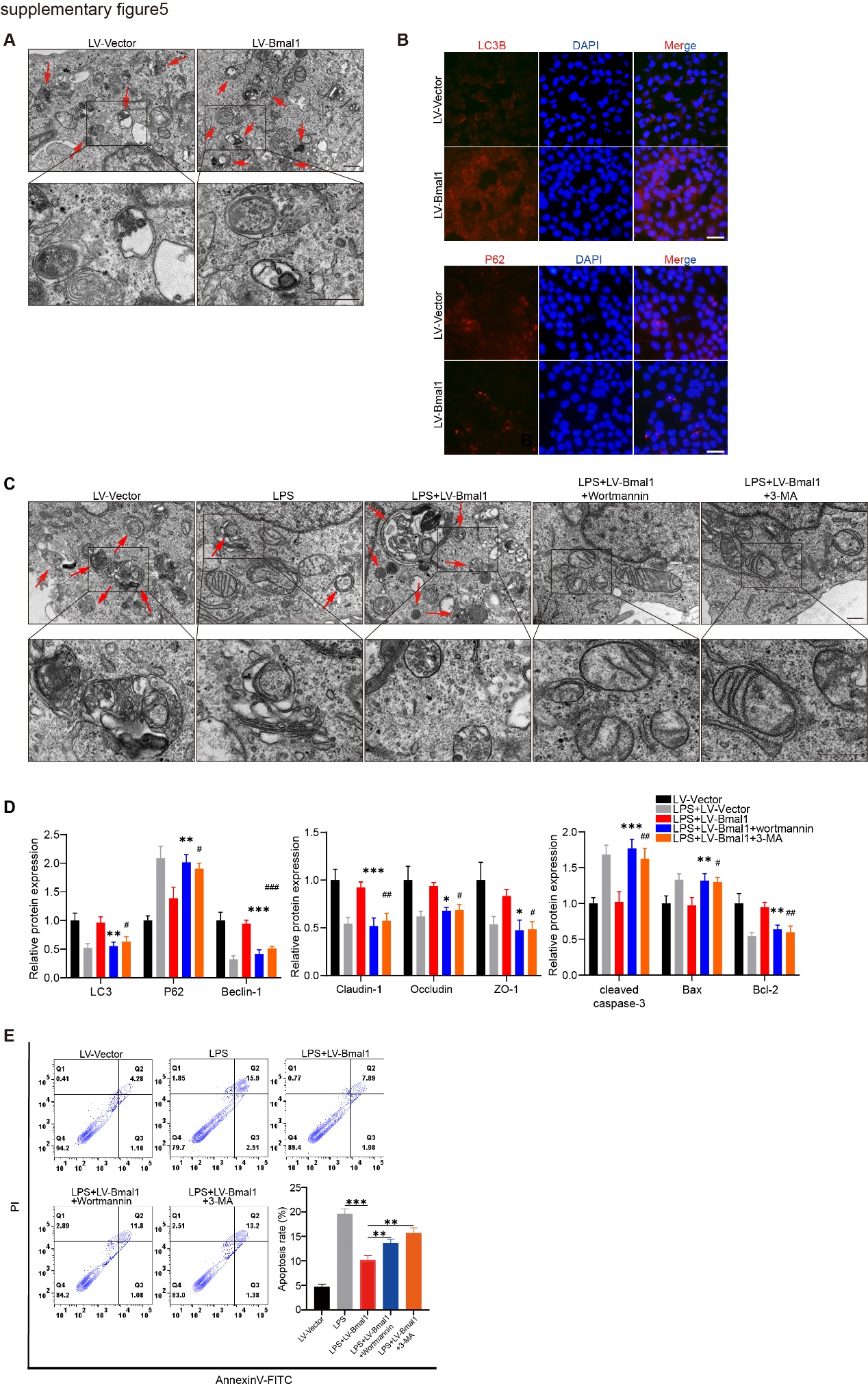


**Supplementary Fig. 5 Bmal1 regulated intestinal barrier function by modulating autophagy**

**A** Representative electron microscopy images of the LV-Bmal1 and LV-Vector groups. Arrows denote autophagosomes. Scale bars: 500 nm.

**B** Representative images of immunofluorescent staining of LC3B and P62 in the LV-Bmal1 and LV-Vector groups. Scale bars: 50 µm.

**C** Representative electron microscopy images in each group. Arrows denote autophagosomes. Scale bars: 500 nm.

**D** Quantitative analysis of the protein levels of LC3, P62, Beclin-1, Claudin-1, Occludin, ZO-1, cleaved caspase-3, Bax, and Bcl-2 in each group. *p < 0.05, **p < 0.01, ***p < 0.001 vs LPS + LV-Bmal1 group,  ^#^ p < 0.05, ^##^ p < 0.01, ^###^ p < 0.001 vs LPS + LV-Bmal1 group.

**E** Representative dot plots of groups of cells double-stained by annexin V-FITC/PI with detection by flow cytometry. Data are presented as the mean ± SD. **p < 0.01, ***p < 0.001.


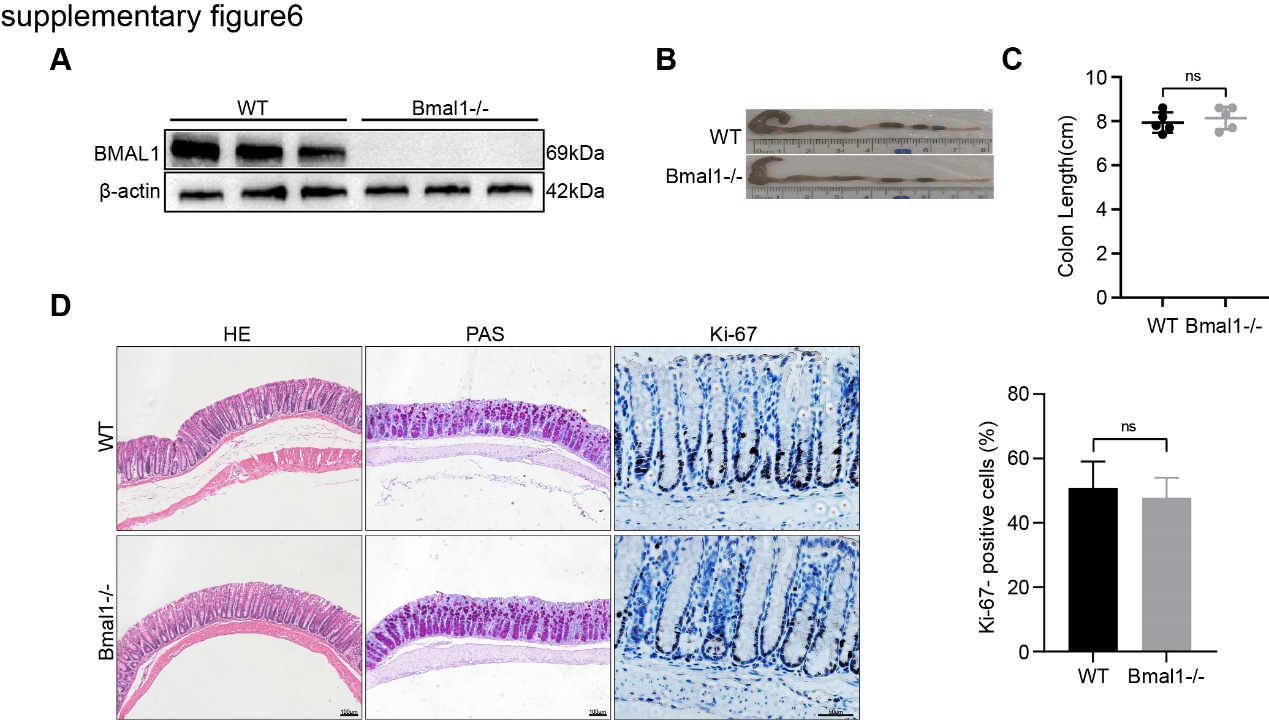


**Supplementary Fig. 6 Bmal1 knockout has no significant effect on mouse colonic function at baseline**

**A** Western blot analysis of Bmal1 protein levels in whole colon tissue samples from 2 groups of mice (WT and Bmal1-/-).

**B** and **C** Colon length and its quantitative analysis in the 2 groups of mice. n = 5 mice per group.

**D** Representative images of hematoxylin and eosin, PAS, and Ki-67 staining of colonic sections from the 2 groups of mice. ns : no significance. Scale bars: 100 μm in HE, PAS images, 50μm in Ki-67 staining images.


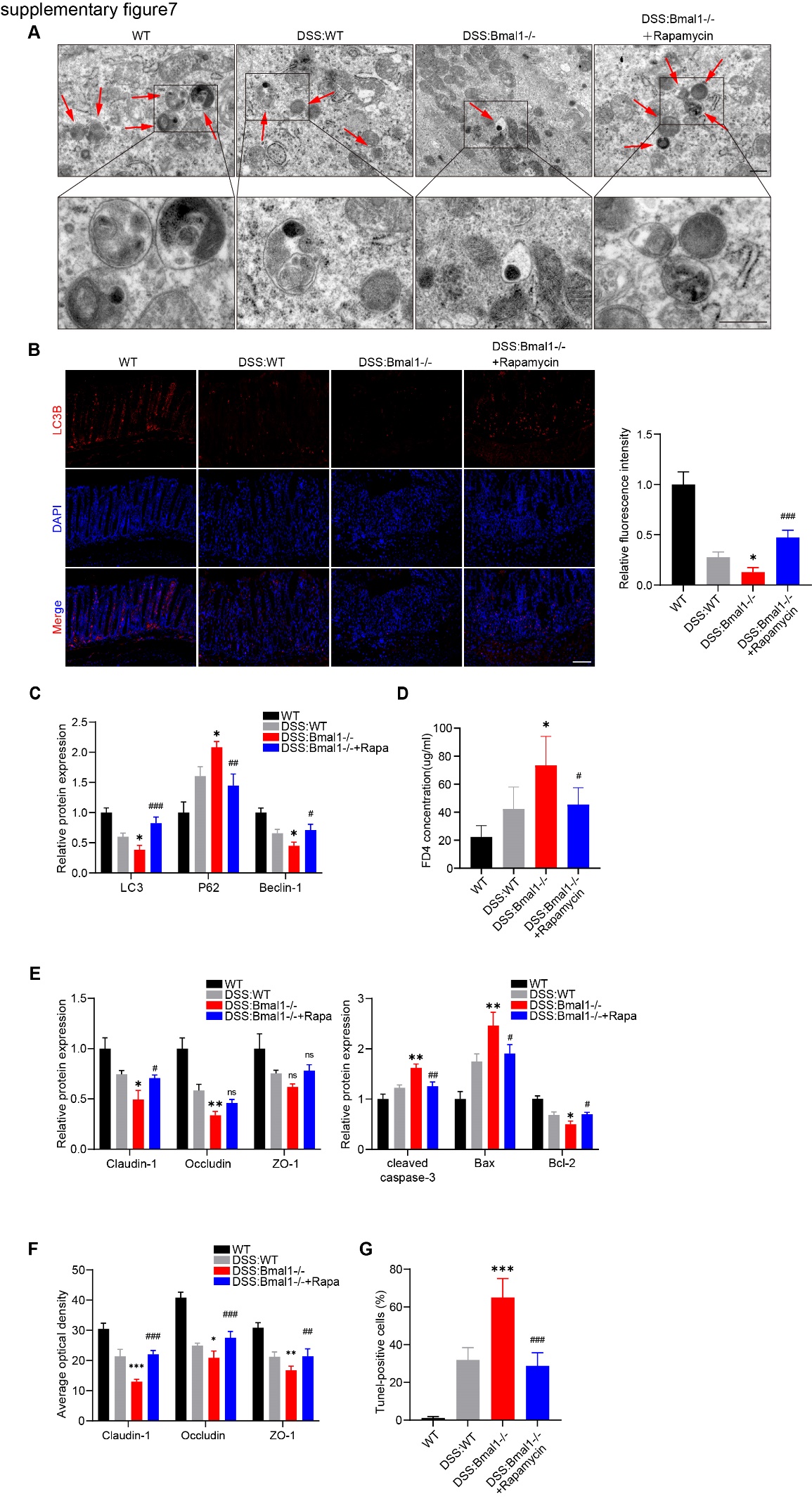


**Supplementary Fig. 7 Bmal1 deficiency exacerbates DSS-induced colitis by inhibiting autophagy**

**A** Representative electron microscopy images of colon tissues from each group of mice. Arrows indicate autophagosomes. Scale bars: 500 nm.

**B** Immunofluorescent staining of LC3B in colon sections from each group of mice. Scale bars: 100 μm.

**C** Quantitative analysis of the protein levels of LC3, P62 and Beclin-1 in each group.

**D** Intestinal permeability was assessed by measuring serum FITC-dextran concentrations in each group of mice. n = 5 mice per group.

**E** Quantitative analysis of the protein levels of Claudin-1, Occludin, ZO-1, cleaved caspase-3, Bax, and Bcl-2 in each group.

**F** Quantitative analysis of the immunohistochemical staining results of each group.

**G** Quantitative analysis of the TUNEL staining results of each group.

ns: no significance, *p < 0.05, **p < 0.01, ***p < 0.001 vs DSS:WT group, ns: no significance, ^#^ p < 0.05, ^##^ p < 0.01, ^###^ p < 0.001 vs DSS:Bmal1-/- group.
